# 7-Chloro-4-[(*E*)-*N*′-(4-fluoro­benzyl­idene)hydrazin­yl]quinoline monohydrate

**DOI:** 10.1107/S1600536809053367

**Published:** 2009-12-16

**Authors:** Marcus V. N. de Souza, R. Alan Howie, Edward R. T. Tiekink, James L. Wardell, Solange M. S. V. Wardell

**Affiliations:** a Instituto de Tecnologia em Farmacos, Fundação Oswaldo Cruz (FIOCRUZ), FarManguinhos, Rua Sizenando Nabuco, 100, Manguinhos, 21041-250 Rio de Janeiro, RJ, Brazil; b Department of Chemistry, University of Aberdeen, Old Aberdeen AB15 5NY, Scotland; c Department of Chemistry, University of Malaya, 50603 Kuala Lumpur, Malaysia; d Centro de Desenvolvimento Tecnológico em Saúde (CDTS), Fundação Oswaldo Cruz (FIOCRUZ), Casa Amarela, Campus de Manguinhos, Av. Brasil 4365, 21040-900 Rio de Janeiro, RJ, Brazil; e CHEMSOL, 1 Harcourt Road, Aberdeen AB15 5NY, Scotland

## Abstract

The mol­ecule of the title hydrate, C_16_H_11_ClFN_3_·H_2_O, is slightly twisted, as  indicated by the dihedral angle of 9.55 (10)° formed between the quinoline ring system and the benzene ring. The conformation about the C=N double bond is *E*, and the amine-H atom is oriented towards the quinoline residue. In the crystal structure, the water mol­ecule accepts an N—H⋯O and makes two O—H⋯N_quinoline_ hydrogen bonds, generating a two-dimensional array in the *ab* plane, which is further stabilized by C—H⋯O inter­actions. The most significant contacts between layers are of the type C—H⋯F.

## Related literature

For background information on the pharmacological activity of quinoline derivatives, see: Elslager *et al.* (1969 ▶); Font *et al.* (1997 ▶); Kaminsky & Meltzer (1968 ▶); Musiol *et al.* (2006 ▶); Nakamura *et al.* (1999 ▶); Palmer *et al.* (1993 ▶); Ridley (2002 ▶); Sloboda *et al.* (1991 ▶); Tanenbaum & Tuffanelli (1980 ▶); Warshakoon *et al.* (2006 ▶). For recent studies into quinoline-based anti-malarials, see: Andrade *et al.* (2007 ▶); Cunico *et al.* (2006 ▶); da Silva *et al.* (2003 ▶); de Souza *et al.* (2005 ▶). For crystallographic studies on mol­ecules related to the title compound, see: Kaiser *et al.* (2009 ▶); de Souza *et al.* (2009 ▶); de Ferreira *et al.* (2009 ▶). For the synthesis, see: Pellerano *et al.* (1976 ▶).
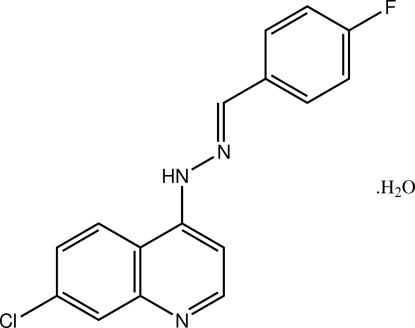


## Experimental

### 

#### Crystal data

C_16_H_11_ClFN_3_·H_2_O
*M* *_r_* = 317.74
Monoclinic, 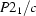

*a* = 3.7795 (2) Å
*b* = 15.4188 (11) Å
*c* = 24.8576 (16) Å
β = 90.286 (4)°
*V* = 1448.57 (16) Å^3^
*Z* = 4
Mo *K*α radiation
μ = 0.28 mm^−1^
*T* = 120 K
0.90 × 0.04 × 0.04 mm

#### Data collection

Nonius KappaCCD area-detector diffractometer
Absorption correction: multi-scan (*SADABS*; Sheldrick, 2007 ▶) *T*_min_ = 0.614, *T*_max_ = 0.746
19494 measured reflections
3291 independent reflections
2009 reflections with *I* > 2σ(*I*)
*R*_int_ = 0.098

#### Refinement

*R*[*F* ^2^ > 2σ(*F* ^2^)] = 0.059
*wR*(*F* ^2^) = 0.131
*S* = 1.04
3291 reflections
205 parameters
3 restraints
H atoms treated by a mixture of independent and constrained refinement
Δρ_max_ = 0.33 e Å^−3^
Δρ_min_ = −0.37 e Å^−3^

### 

Data collection: *COLLECT* (Hooft, 1998 ▶); cell refinement: *DENZO* (Otwinowski & Minor, 1997 ▶) and *COLLECT*; data reduction: *DENZO* and *COLLECT*; program(s) used to solve structure: *SHELXS97* (Sheldrick, 2008 ▶); program(s) used to refine structure: *SHELXL97* (Sheldrick, 2008 ▶); molecular graphics: *DIAMOND* (Brandenburg, 2006 ▶); software used to prepare material for publication: *publCIF* (Westrip, 2009 ▶).

## Supplementary Material

Crystal structure: contains datablocks global, I. DOI: 10.1107/S1600536809053367/lh2970sup1.cif

Structure factors: contains datablocks I. DOI: 10.1107/S1600536809053367/lh2970Isup2.hkl

Additional supplementary materials:  crystallographic information; 3D view; checkCIF report

## Figures and Tables

**Table 1:** Hydrogen-bond geometry (Å, °)

*D*—H⋯*A*	*D*—H	H⋯*A*	*D*⋯*A*	*D*—H⋯*A*
O1w—H1w⋯N1^i^	0.84 (2)	2.28 (2)	2.999 (3)	144 (2)
O1w—H2w⋯N1^ii^	0.85 (2)	1.93 (2)	2.761 (3)	166 (3)
N2—H2n⋯O1w^iii^	0.88	2.01	2.865 (3)	165
C5—H5⋯O1w^iii^	0.95	2.45	3.379 (3)	164
C10—H10⋯O1w^iii^	0.95	2.50	3.302 (3)	142
C1—H1⋯F1^iv^	0.95	2.56	3.399 (3)	147
C6—H6⋯F1^v^	0.95	2.56	3.477 (3)	161

Symmetry codes: (i) 
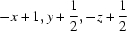
; (ii) 
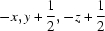
; (iii) 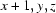; (iv) 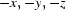; (v) 
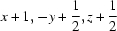
.

**Fig. 1.**
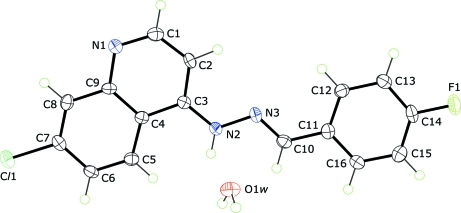
The molecular structure of both components comprising the asymmetric unit of (I) showing the atom-labelling scheme and displacement ellipsoids at the 50% probability level.

**Fig. 2.**
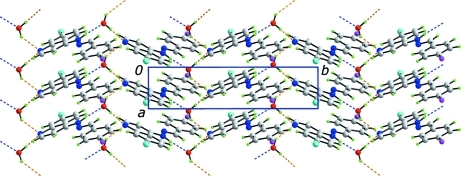
A view of the 2-D supramolecular array in (I) showing the O–H···N and N–H···O hydrogen bonds as orange and blue dashed lines, respectively. Colour code: Cl, cyan; F, pink; O, red; N, blue; C, grey; and H, green.

**Fig. 3.**
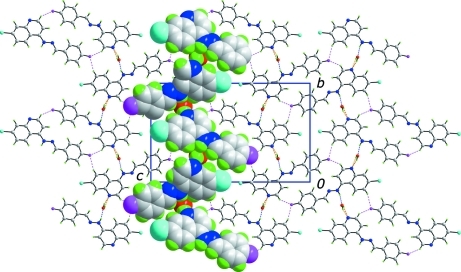
A view in projection along the a axis of the unit-cell contents in (I) showing the stacking of layers along the c direction. The O–H···N and N–H···O hydrogen bonds are shown as orange and blue dashed lines, respectively, and the C–H···F contacts are represented by pink dashed lines. One of the 2-D arrays, as shown in Fig. 2, has been highlighted in space-filling mode. Colour code: Cl, cyan; F, pink; O, red; N, blue; C, grey; and H, green.

## supplementary crystallographic information

### Comment

The title compound, crystallized as a hydrate, (I), was prepared as part of continuing studies designed to develop antimalarial compounds based on the quinoline nucleus (Andrade *et al.*, 2007; Cunico *et al.*, 2006; da Silva *et al.*, 2003; de Souza *et al.*,  2005). The systematic examination of quinoline derivatives comes about owing to the fact that the majority of antimalarial agents, including chloroquine (Tanenbaum & Tuffanelli, 1980), mefloquine (Palmer *et al.*, 1993), primaquine (Elslager *et al.*, 1969) and amodiaquine (Ridley, 2002), have a quinoline ring substructure, the mainstay of malaria chemotherapy for much of the past 40 years (Font *et al.*, 1997; Kaminsky & Meltzer, 1968; Musiol *et al.*, 2006; Nakamura *et al.*, 1999; Sloboda *et al.*, 1991; Warshakoon *et al.*, 2006). Allied with these investigations are structural studies aimed at elucidating systematic structural trends in these molecules (Kaiser *et al.* 2009; de Souza *et al.* 2009; de Ferreira *et al.* 2009).

The molecule in (I), Fig. 1, features an effectively planar quinoline residue (maximum deviations of 0.018 (2) Å for atom C4 and -0.025 (2) Å for atom C2) which forms a dihedral angle of 9.55 (10) ° with the C11–C16 benzene ring. Twists in the molecule are evident about the N2–C3 and C10–C11 bonds as seen in the values of the N3–N2–C3–C2 and N3–C10–C11—C12 torsion angles of 6.9 (4) and -6.6 (4) °, respectively. As observed in related systems, the amine-H is orientated over the quinoline residue (Kaiser *et al.* 2009; de Souza *et al.* 2009; de Ferreira *et al.*, 2009). The conformation about the N3═C10 double bond is *E*. The molecule crystallizes as a hydrate and the latter species is pivotal in stabilizing the crystal structure. Thus, the water-H atoms form donor O–H···N hydrogen bonds to quinoline-N atoms derived from two molecules. At the same time, the water-O atom accepts a N–H···O hydrogen bond from the amine-N2 of another molecule. Thus, the water molecule provides links between three molecules, leading to the formation of a 2-D array, Fig. 2 and Table 1. The resultant layer in the *ab* plane is further stabilized by C–H···O interactions, Table 1, and weak π···π contacts [ring centroid(N1,C1—C4,C9)···ring centroid(C4–C9)^i^ = 3.7070 (14) Å, dihedral angle = 1.45 (11) ° for *i*: -1 + *x*, *y*, *z*]. Layers stack along the *c* direction with the most significant contacts between layers being of the type C–H···F whereby the fluoride is bifurcated, Table 1 and Fig. 3.

### Experimental

A solution of 7-chloro-4-hydrazinoquinoline (0.20 g, 1.0 mmol) and 4-fluorobenzaldehyde (0.15 g, 1.2 mmol) in EtOH (5 ml) was maintained at room temperature overnight and rotary evaporated. The solid residue, was washed with cold Et_2_O (3 *x* 10 ml) and recrystallized from EtOH m.pt. 518–519 K, lit. value 518 K (Pellerano *et al.*, 1976), yield 74%. The sample for the X-ray study was slowly grown from moist EtOH and was found to be the monohydrate. ^1^H NMR (400 MHz, DMSO-d_6_) δ: 7.28–7.32 (3*H*, m), 7.54 (1*H*, d, J = 8.4 Hz), 7.84–7.88 (3*H*, m), 8.34–8.40 (3*H*, m), 11.3 (1*H*, br.s, NH). MS/ESI: [*M*^+.^ - H]: 298. IR ν_max_ (cm^-1^; KBr disc): 3232 (N–H), 1585 (C═N), 817 (C–F).

### Refinement

The amine- and C-bound H atoms were geometrically placed (N–H = 0.88 Å and C–H = 0.95 Å) and refined as riding with *U_iso_*(H) = 1.2*U_eq_*(C). The water-bound H atoms were located from a difference map and refined (O–H = 0.84 (1) Å) with *U_iso_*(H) = 1.5*U_eq_*(O).

### Crystal data

**Table:**

C_16_H_11_ClFN_3_·H_2_O	*F*(000) = 656
*M**_r_* = 317.74	*D*_x_ = 1.457 Mg m^−^^3^
Monoclinic, *P*2_1_/*c*	Mo *K*α radiation, λ = 0.71073 Å
Hall symbol: -P 2ybc	Cell parameters from 13530 reflections
*a* = 3.7795 (2) Å	θ = 2.9–27.5°
*b* = 15.4188 (11) Å	µ = 0.28 mm^−^^1^
*c* = 24.8576 (16) Å	*T* = 120 K
β = 90.286 (4)°	Needle, colourless
*V* = 1448.57 (16) Å^3^	0.90 × 0.04 × 0.04 mm
*Z* = 4	

### Data collection

**Table:**

Enraf–Nonius KappaCCD area-detector diffractometer	3291 independent reflections
Radiation source: Enraf Nonius FR591 rotating anode	2009 reflections with *I* > 2σ(*I*)
10 cm confocal mirrors	*R*_int_ = 0.098
Detector resolution: 9.091 pixels mm^-1^	θ_max_ = 27.5°, θ_min_ = 3.1°
φ and ω scans	*h* = −4→4
Absorption correction: multi-scan (*SADABS*; Sheldrick, 2007)	*k* = −20→19
*T*_min_ = 0.614, *T*_max_ = 0.746	*l* = −32→32
19494 measured reflections	

### Refinement

**Table:**

Refinement on *F*^2^	Primary atom site location: structure-invariant direct methods
Least-squares matrix: full	Secondary atom site location: difference Fourier map
*R*[*F*^2^ > 2σ(*F*^2^)] = 0.059	Hydrogen site location: inferred from neighbouring sites
*wR*(*F*^2^) = 0.131	H atoms treated by a mixture of independent and constrained refinement
*S* = 1.04	*w* = 1/[σ^2^(*F*_o_^2^) + (0.0463*P*)^2^ + 0.5902*P*] where *P* = (*F*_o_^2^ + 2*F*_c_^2^)/3
3291 reflections	(Δ/σ)_max_ < 0.001
205 parameters	Δρ_max_ = 0.33 e Å^−^^3^
3 restraints	Δρ_min_ = −0.37 e Å^−^^3^

### Special details

**Table:**

Geometry. All s.u.'s (except the s.u. in the dihedral angle between two l.s. planes) are estimated using the full covariance matrix. The cell s.u.'s are taken into account individually in the estimation of s.u.'s in distances, angles and torsion angles; correlations between s.u.'s in cell parameters are only used when they are defined by crystal symmetry. An approximate (isotropic) treatment of cell s.u.'s is used for estimating s.u.'s involving l.s. planes.
Refinement. Refinement of *F*^2^ against ALL reflections. The weighted *R*-factor *wR* and goodness of fit *S* are based on *F*^2^, conventional *R*-factors *R* are based on *F*, with *F* set to zero for negative *F*^2^. The threshold expression of *F*^2^ > 2σ(*F*^2^) is used only for calculating *R*-factors(gt) *etc*. and is not relevant to the choice of reflections for refinement. *R*-factors based on *F*^2^ are statistically about twice as large as those based on *F*, and *R*- factors based on ALL data will be even larger.

### Fractional atomic coordinates and isotropic or equivalent isotropic displacement parameters (Å^2^)

**Table:**

	*x*	*y*	*z*	*U*_iso_*/*U*_eq_	
Cl1	0.85154 (18)	−0.01273 (5)	0.43677 (3)	0.0319 (2)	
F1	0.1997 (4)	0.22958 (11)	−0.11624 (6)	0.0367 (5)	
N1	0.3942 (5)	−0.13713 (14)	0.26095 (9)	0.0214 (5)	
N2	0.6585 (5)	0.08516 (14)	0.17189 (8)	0.0221 (5)	
H2N	0.7847	0.1268	0.1867	0.027*	
N3	0.5476 (5)	0.09163 (15)	0.11914 (8)	0.0212 (5)	
C1	0.3270 (7)	−0.12969 (18)	0.20855 (11)	0.0225 (6)	
H1	0.2176	−0.1776	0.1911	0.027*	
C2	0.4043 (6)	−0.05737 (18)	0.17733 (10)	0.0210 (6)	
H2	0.3436	−0.0566	0.1402	0.025*	
C3	0.5701 (6)	0.01357 (17)	0.20052 (10)	0.0178 (6)	
C4	0.6457 (6)	0.01003 (16)	0.25750 (10)	0.0176 (6)	
C5	0.8024 (6)	0.07868 (18)	0.28696 (11)	0.0210 (6)	
H5	0.8680	0.1303	0.2687	0.025*	
C6	0.8613 (6)	0.07213 (18)	0.34118 (10)	0.0220 (6)	
H6	0.9655	0.1189	0.3605	0.026*	
C7	0.7663 (7)	−0.00432 (18)	0.36790 (11)	0.0218 (6)	
C8	0.6151 (6)	−0.07217 (18)	0.34132 (10)	0.0214 (6)	
H8	0.5531	−0.1233	0.3604	0.026*	
C9	0.5501 (6)	−0.06654 (16)	0.28533 (10)	0.0179 (6)	
C10	0.6205 (7)	0.16293 (18)	0.09495 (11)	0.0219 (6)	
H10	0.7507	0.2064	0.1136	0.026*	
C11	0.5085 (7)	0.17884 (18)	0.03962 (11)	0.0216 (6)	
C12	0.3483 (7)	0.11448 (18)	0.00825 (11)	0.0231 (6)	
H12	0.3087	0.0586	0.0232	0.028*	
C13	0.2471 (7)	0.13101 (18)	−0.04411 (11)	0.0237 (6)	
H13	0.1409	0.0871	−0.0656	0.028*	
C14	0.3041 (7)	0.21294 (19)	−0.06453 (11)	0.0256 (7)	
C15	0.4572 (7)	0.27824 (19)	−0.03525 (11)	0.0269 (7)	
H15	0.4911	0.3342	−0.0504	0.032*	
C16	0.5614 (7)	0.26028 (18)	0.01715 (11)	0.0232 (6)	
H16	0.6708	0.3044	0.0380	0.028*	
O1W	0.0566 (5)	0.23633 (12)	0.20098 (8)	0.0287 (5)	
H1W	0.262 (3)	0.2503 (17)	0.2111 (12)	0.043*	
H2W	−0.086 (5)	0.2775 (13)	0.2069 (12)	0.043*	

### Atomic displacement parameters (Å^2^)

**Table:**

	*U*^11^	*U*^22^	*U*^33^	*U*^12^	*U*^13^	*U*^23^
Cl1	0.0381 (4)	0.0386 (5)	0.0191 (4)	−0.0007 (3)	−0.0065 (3)	−0.0001 (3)
F1	0.0475 (10)	0.0422 (11)	0.0205 (9)	0.0086 (8)	−0.0082 (8)	0.0037 (8)
N1	0.0218 (12)	0.0225 (13)	0.0200 (13)	0.0000 (10)	−0.0006 (9)	−0.0006 (10)
N2	0.0269 (12)	0.0218 (13)	0.0177 (12)	−0.0046 (10)	−0.0046 (9)	0.0001 (10)
N3	0.0219 (12)	0.0265 (14)	0.0153 (12)	0.0018 (10)	−0.0022 (9)	0.0005 (10)
C1	0.0185 (14)	0.0204 (16)	0.0286 (17)	0.0005 (11)	−0.0024 (12)	−0.0026 (13)
C2	0.0204 (14)	0.0270 (16)	0.0156 (14)	0.0007 (12)	−0.0052 (11)	−0.0005 (12)
C3	0.0150 (13)	0.0189 (15)	0.0195 (14)	0.0014 (11)	−0.0008 (10)	−0.0011 (12)
C4	0.0151 (13)	0.0168 (14)	0.0209 (14)	0.0033 (11)	−0.0002 (10)	−0.0018 (12)
C5	0.0204 (14)	0.0189 (15)	0.0237 (15)	0.0012 (11)	0.0001 (11)	−0.0018 (12)
C6	0.0242 (15)	0.0210 (16)	0.0208 (15)	−0.0003 (12)	−0.0035 (11)	−0.0059 (12)
C7	0.0204 (14)	0.0276 (17)	0.0175 (14)	0.0030 (12)	−0.0016 (11)	−0.0034 (12)
C8	0.0198 (14)	0.0223 (16)	0.0220 (15)	0.0050 (12)	−0.0007 (11)	0.0046 (12)
C9	0.0154 (13)	0.0149 (14)	0.0233 (15)	0.0003 (11)	−0.0026 (10)	−0.0030 (12)
C10	0.0222 (15)	0.0215 (16)	0.0221 (16)	0.0019 (12)	0.0000 (11)	−0.0046 (13)
C11	0.0185 (14)	0.0253 (16)	0.0209 (15)	0.0030 (12)	0.0004 (11)	0.0010 (12)
C12	0.0261 (15)	0.0194 (15)	0.0238 (16)	−0.0004 (12)	0.0010 (12)	−0.0001 (12)
C13	0.0240 (15)	0.0241 (16)	0.0229 (16)	0.0009 (12)	−0.0018 (12)	−0.0045 (13)
C14	0.0273 (15)	0.0350 (18)	0.0145 (14)	0.0051 (13)	−0.0025 (11)	0.0012 (13)
C15	0.0265 (15)	0.0256 (17)	0.0287 (17)	0.0025 (13)	−0.0004 (12)	0.0043 (13)
C16	0.0244 (15)	0.0240 (16)	0.0213 (15)	−0.0016 (12)	−0.0012 (11)	−0.0037 (13)
O1W	0.0255 (11)	0.0227 (11)	0.0377 (13)	−0.0003 (9)	−0.0020 (9)	−0.0045 (9)

### Geometric parameters (Å, °)

**Table:**

Cl1—C7	1.745 (3)	C6—H6	0.9500
F1—C14	1.367 (3)	C7—C8	1.362 (4)
N1—C1	1.331 (3)	C8—C9	1.414 (3)
N1—C9	1.377 (3)	C8—H8	0.9500
N2—C3	1.356 (3)	C10—C11	1.458 (4)
N2—N3	1.378 (3)	C10—H10	0.9500
N2—H2N	0.8800	C11—C16	1.389 (4)
N3—C10	1.284 (3)	C11—C12	1.398 (4)
C1—C2	1.390 (4)	C12—C13	1.379 (4)
C1—H1	0.9500	C12—H12	0.9500
C2—C3	1.385 (4)	C13—C14	1.379 (4)
C2—H2	0.9500	C13—H13	0.9500
C3—C4	1.445 (3)	C14—C15	1.369 (4)
C4—C5	1.415 (4)	C15—C16	1.387 (4)
C4—C9	1.416 (4)	C15—H15	0.9500
C5—C6	1.369 (3)	C16—H16	0.9500
C5—H5	0.9500	O1W—H1W	0.841 (10)
C6—C7	1.401 (4)	O1W—H2W	0.845 (10)
			
C1—N1—C9	116.2 (2)	C7—C8—H8	120.0
C3—N2—N3	118.9 (2)	C9—C8—H8	120.0
C3—N2—H2N	120.5	N1—C9—C8	117.2 (2)
N3—N2—H2N	120.5	N1—C9—C4	123.6 (2)
C10—N3—N2	116.3 (2)	C8—C9—C4	119.2 (2)
N1—C1—C2	125.2 (3)	N3—C10—C11	121.6 (2)
N1—C1—H1	117.4	N3—C10—H10	119.2
C2—C1—H1	117.4	C11—C10—H10	119.2
C3—C2—C1	119.8 (2)	C16—C11—C12	118.7 (2)
C3—C2—H2	120.1	C16—C11—C10	119.3 (2)
C1—C2—H2	120.1	C12—C11—C10	122.0 (3)
N2—C3—C2	122.4 (2)	C13—C12—C11	120.8 (3)
N2—C3—C4	119.8 (2)	C13—C12—H12	119.6
C2—C3—C4	117.7 (2)	C11—C12—H12	119.6
C5—C4—C9	118.5 (2)	C12—C13—C14	118.3 (3)
C5—C4—C3	124.0 (2)	C12—C13—H13	120.9
C9—C4—C3	117.4 (2)	C14—C13—H13	120.9
C6—C5—C4	121.3 (3)	F1—C14—C15	118.8 (3)
C6—C5—H5	119.3	F1—C14—C13	118.3 (2)
C4—C5—H5	119.3	C15—C14—C13	123.0 (3)
C5—C6—C7	119.2 (2)	C14—C15—C16	118.0 (3)
C5—C6—H6	120.4	C14—C15—H15	121.0
C7—C6—H6	120.4	C16—C15—H15	121.0
C8—C7—C6	121.6 (2)	C15—C16—C11	121.2 (3)
C8—C7—Cl1	119.6 (2)	C15—C16—H16	119.4
C6—C7—Cl1	118.8 (2)	C11—C16—H16	119.4
C7—C8—C9	120.0 (2)	H1W—O1W—H2W	110.0 (16)
			
C3—N2—N3—C10	176.0 (2)	C7—C8—C9—N1	−179.5 (2)
C9—N1—C1—C2	0.5 (4)	C7—C8—C9—C4	0.3 (4)
N1—C1—C2—C3	1.3 (4)	C5—C4—C9—N1	179.6 (2)
N3—N2—C3—C2	6.9 (4)	C3—C4—C9—N1	0.7 (4)
N3—N2—C3—C4	−172.4 (2)	C5—C4—C9—C8	−0.2 (3)
C1—C2—C3—N2	178.6 (2)	C3—C4—C9—C8	−179.1 (2)
C1—C2—C3—C4	−2.0 (4)	N2—N3—C10—C11	−178.2 (2)
N2—C3—C4—C5	1.6 (4)	N3—C10—C11—C16	173.3 (2)
C2—C3—C4—C5	−177.7 (2)	N3—C10—C11—C12	−6.6 (4)
N2—C3—C4—C9	−179.5 (2)	C16—C11—C12—C13	0.5 (4)
C2—C3—C4—C9	1.1 (3)	C10—C11—C12—C13	−179.5 (2)
C9—C4—C5—C6	−0.1 (4)	C11—C12—C13—C14	−0.8 (4)
C3—C4—C5—C6	178.7 (2)	C12—C13—C14—F1	−179.2 (2)
C4—C5—C6—C7	0.4 (4)	C12—C13—C14—C15	0.2 (4)
C5—C6—C7—C8	−0.3 (4)	F1—C14—C15—C16	180.0 (2)
C5—C6—C7—Cl1	178.65 (19)	C13—C14—C15—C16	0.5 (4)
C6—C7—C8—C9	0.0 (4)	C14—C15—C16—C11	−0.8 (4)
Cl1—C7—C8—C9	−178.98 (18)	C12—C11—C16—C15	0.2 (4)
C1—N1—C9—C8	178.3 (2)	C10—C11—C16—C15	−179.7 (2)
C1—N1—C9—C4	−1.5 (4)		

### Hydrogen-bond geometry (Å, °)

**Table:**

*D*—H···*A*	*D*—H	H···*A*	*D*···*A*	*D*—H···*A*
O1w—H1w···N1^i^	0.843 (16)	2.28 (2)	2.999 (3)	144 (2)
O1w—H2w···N1^ii^	0.85 (2)	1.93 (2)	2.761 (3)	166 (3)
N2—H2n···O1w^iii^	0.88	2.01	2.865 (3)	165
C5—H5···O1w^iii^	0.95	2.45	3.379 (3)	164
C10—H10···O1w^iii^	0.95	2.50	3.302 (3)	142
C1—H1···F1^iv^	0.95	2.56	3.399 (3)	147
C6—H6···F1^v^	0.95	2.56	3.477 (3)	161

Symmetry codes: (i) −*x*+1, *y*+1/2, −*z*+1/2; (ii) −*x*, *y*+1/2, −*z*+1/2; (iii) *x*+1, *y*, *z*; (iv) −*x*, −*y*, −*z*; (v) *x*+1, −*y*+1/2, *z*+1/2.
